# Clinical Symptoms Contributing to Zenker's Diverticulum Repair: A Retrospective Review

**DOI:** 10.7759/cureus.22369

**Published:** 2022-02-18

**Authors:** Jhon F Martinez Paredes, Razan Al Fakir, Amy L Rutt

**Affiliations:** 1 Department of Surgery, The University of Texas Rio Grande Valley, McAllen, USA; 2 Department of Otolaryngology, Auburn University, Auburn, USA; 3 Otolaryngology, Mayo Clinic, Jacksonville, USA

**Keywords:** swallowing, quality of life, dysphonia, dysphagia, zenker's diverticulum

## Abstract

Objective: Zenker's diverticulum (ZD) is usually associated with dysphagia and other symptoms due to the interrelated functions of several systems. Surgical management of ZD is effective for all sizes of diverticula, but not all patients decide to undergo surgery. The purpose of this study was to determine the relationship between clinical presentation and patients' decision to undergo surgical repair.

Subjects and methods: This is a retrospective study including 165 patients with ZD treated over the last 11 years. Data collection includes patients' chief complaints and symptoms, medical history, findings on radiologic swallow evaluations, and patients' decision to undergo surgery. Pearson correlation and logistic regression analysis were performed.

Results: Among our cohort, dysphagia was the most prevalent symptom (89.1%), followed by cough (65.5%) and regurgitation (58.8%). Dysphonia was prevalent among patients with a small-sized ZD. Our logistic regression model showed that patients' decision to undergo surgical repair could be predicted by diverticula size (β=1.10, p=0.002) and the presence of dysphagia (β=1.91, p=0.005), cough (β=1.01, p=0.042), and dysphonia (β=-1.37, p=0.024).

Conclusion: Patients' decision to undergo surgery usually involves interrelated factors, including symptomatic burden, presence of comorbidities, and recommendation of the surgeon. This study has identified that diverticula size and the presence of dysphagia, cough, and dysphonia are significant factors influencing decision-making for surgical repair in patients with ZD.

## Introduction

The functioning of the cricopharyngeus muscle, which is strategically placed between the pharynx and the esophagus, is vital for health. Zenker's diverticulum (ZD) is an esophageal disorder believed to be caused by cricopharyngeal muscle dysfunction [1-3]. This pathology has a slight male predominance, and its peak incidence is between the seventh and ninth decades of life. The incidental rate is two per 100,000, with prevalence between 0.01% and 0.11% [4]. Clinical presentation and modified barium swallow study (MBSS) are the mainstay in the diagnosis of ZD [5,6].

Surgical management of ZD is recommended for symptomatic patients. These symptoms include regurgitation, choking, chronic cough, globus, weight loss, and, less commonly, dysphonia. Surgical treatment includes one of the following procedures: open transcervical diverticulectomy, endoscopic laser diverticulectomy, flexible endoscopic diverticulectomy, and endoscopic stapler-assisted diverticulectomy. A meta-analysis published in 2019 by Howell et al. established no differences in outcomes between the different approaches and demonstrated that any surgical intervention yields a significant effect on postoperative symptom relief [7].

The main criteria for surgical repair depend on clinical variables, including age, the presence of dysphagia, the presence of comorbidities, and the size of the diverticula [4,8-10]. Although dysphagia symptoms are usually one of the factors that motivate patients to look for medical care [11], the relationship between clinical presentation and the decision of patients with ZD to undergo surgery is unknown. Understanding these relationships would help in the counseling process of patients' decisions for ZD surgical repair. The purpose of this study was, therefore, to determine a relationship between surgical repair and ZD signs and symptoms. The results can be used to improve clinical insight in treatment planning during preoperative and postoperative counseling.

## Materials and methods

Institutional review board approval was obtained before the commencement of this study. We performed a retrospective chart review of the electronic medical records (EMR) of 208 patients with an associated ICD-9 or ICD-10 code for ZD (ICD-9: 530.6, acquired esophageal diverticulum; ICD-10: K22.5, diverticulum of the esophagus, acquired) that presented for a clinical evaluation in the Otolaryngology Outpatient clinic at Mayo Clinic from January 2009 to April 2020 (Jacksonville, FL, and Rochester, MN, locations). From this group, a total of 165 patients met the following inclusion criteria: (i) having a ZD diagnosis with an adequate radiologic evaluation (MBSS or esophagram) and (ii) having complete medical data of initial medical evaluation and surgical intervention. Patients without radiologic evaluation and incomplete data of clinical symptoms at initial assessment and surgical ZD repair information were excluded.

Chart review of EMR included demographic information, clinical symptoms, comorbidities, radiologic evaluations (MBSS or esophagram), and postoperative follow-up visits. The initial medical evaluation included assessment of the main complaint and other symptoms, including dysphagia, odynophagia, dysphonia, cough, choking, regurgitation, aspiration, globus, and weight loss. Diagnosis of dysphonia was confirmed by a laryngologist.

ZD size was measured preoperatively as proposed by van Overbeek and Groote using a radiologic scale [12] and confirmed intraoperatively. The size was stratified into three groups: small (<1 cm), moderate (1-3 cm), and large (>3 cm). Patients were followed after diagnosis to identify their decisions about undergoing a ZD repair. The Research Electronic Data Capture (REDCap) project was used to collect the data.

Statistical analyses were performed using SPSS version 25 (IBM Corporation, Armonk, NY, USA). Standard descriptive statistics were obtained and presented as percentages and mean ± standard deviations (SD). Pearson correlation analysis and logistic regression were performed to evaluate the association of medical history, presenting symptoms, and ZD size (independent variables) and the decision to undergo a surgical repair (dependent variable). A p-value < 0.05 was considered statistically significant.

## Results

A total of 165 patients were enrolled, of whom 92 (55.7%) were male. The mean age was 73 years (±11), and the average body mass index (BMI) was 26.9 kg/m^2^ (±5.6). Most of our patients had at least one comorbidity. The most frequent comorbidity was gastroesophageal reflux disease (GERD) (65.5%), followed by hypertension (52.1%), hiatal hernia (23%), and esophageal dysmotility (13.9%). Forty-eight patients were diagnosed with a small ZD, 67 with medium size, and 50 with a large-sized diverticulum. Thirty-seven (22.4%) patients had a history of previous ZD repair. From our overall cohort, a total of 131 patients underwent ZD surgical repair. A summary of the patients' demographic and clinical data, including comorbidities, is shown in Table 1.

**Table 1:** Demographic and clinical data of the study population BMI: body mass index; GERD: gastroesophageal reflux disease; OSA: obstructive sleep apnea; SD: standard deviation; ZD: Zenker's diverticulum

Characteristics	n=165
Gender (male), number (%)	92 (55.8)
Age (year), mean (SD)	73 (11)
BMI (kg/m^2^), mean (SD)	26.9 (5.6)
Comorbidities	
GERD, number (%)	108 (65.5)
Hypertension, number (%)	86 (52.1)
Hiatal hernia, number (%)	38 (23)
Esophageal dysmotility, number (%)	23 (13.9)
Diabetes mellitus, number (%)	19 (11.5)
OSA, number (%)	18 (11)
ZD size	
Small (<1 cm)	48 (29.1)
Medium (1–3 cm)	67 (40.6)
Large (>3 cm)	50 (30.3)

The preoperative symptoms and their prevalence were stratified by diverticulum size. Among the overall cohort, dysphagia was the most prevalent symptom (89.1%), followed by cough (65.5%) and regurgitation (58.8%). When analyzing the clinical presentation based on the ZD size, symptoms such as dysphagia, cough, and regurgitation were highly prevalent in the medium-sized diverticulum group. Dysphonia was reported as the fifth most frequent symptom in patients with small ZD and as the least frequent symptom in patients with medium and large pharyngeal pouch. Cough was present in almost two-thirds of cases among the overall cohort.

When evaluating the correlation between different clinical variables, we found a positive statistically significant correlation between the patients' decision to undergo ZD surgical repair and the diverticula size (r=0.39), the presence of dysphagia (r=0.25), and choking (r=0.17). Dysphonia was found to have a negative statistically significant correlation (r=-0.23) (Table 2).

**Table 2:** Pearson correlation between presenting symptoms and the patients' decision to undergo Zenker's diverticulum surgical repair *p-value < 0.05: statistically significant

Clinical variable	r	p-value*
Zenker’s diverticulum size	0.397	0.001
Dysphagia	0.254	0.001
Choking	0.173	0.026
Dysphonia	-0.239	0.002

Logistic regression analysis was performed to evaluate the relationship between the patients' decision-making for surgical repair (dependent variables) and ZD signs and symptoms (independent variables). The logistic regression model showed that patients' decision to undergo a surgical repair could be predicted by diverticula size (β=1.10, p=0.002) and the presence of dysphagia (β=1.91, p=0.005), cough (β=1.01, p=0.042), and dysphonia (β=-1.37, p=0.024). Full details on the logistic regression analysis of predictors are presented in Table 3.

**Table 3:** Logistic regression analysis of the predictors of undergoing Zenker's diverticulum surgical repair *p-value < 0.05: statistically significant

Predictor	β value	Standard error	95% CI	p-value
Zenker’s diverticulum size	1.1	0.35	1.5–5.9	0.002*
Dysphagia	1.91	0.68	1.78–25.8	0.005*
Cough	1.01	0.49	1.04–7.3	0.042*
Choking	0.58	0.54	0.62–5.2	0.28
Dysphonia	-1.37	0.6	0.08–0.83	0.024*

## Discussion

To the best of our knowledge, this is the first study to determine the relationship between surgical repair and ZD signs and symptoms. Our regression model showed that diverticulum size and the presence of dysphagia, cough, and dysphonia were the most common factors that play a role in the decision of patients with ZD to undergo surgical repair. Our results can be used to improve clinical insight in treatment planning during preoperative and postoperative evaluation.

It is well known that surgical repair is effective for all sizes of diverticula [12-14]. However, patients' final decision to undergo surgery usually involves interrelated factors, including symptomatic burden, presence of comorbidities, and recommendation of the surgeon. The relationship between dysphagia characteristics and diverticula size was studied for the first time by Bergeron et al. During their study involving 46 patients with ZD, they noted that the presence of symptoms such as regurgitation, globus, and endoscopically documented preoperative pooling and/or post-swallow hypopharyngeal reflux was associated with a ZD larger than 1 cm [15]. Other studies have demonstrated that larger ZD sizes are associated with more recalcitrant symptoms [13,15]. Our results are consistent with these studies as the prevalence of dysphagia, cough, and regurgitation was higher among those patients with a medium to large ZD. Additionally, our correlation analysis and logistic regression model confirmed that patients with a larger ZD would more likely undergo surgical repair than those with small diverticula. In a large-sized diverticulum, the pouch opening is usually aligned with the pharynx axis with a subsequent prominent deviation of food inside the pouch, leading to more symptomatic scenarios with dysphagia, choking, aspiration episodes, halitosis, neck mass sensation, or even malnourishment [16,17].

Coughing is a nonspecific response to a variety of stimuli usually originating in the pharynx, larynx, or lungs [18]. Thus, cough usually represents one of the main reasons for patients visiting their primary physicians, and its treatment remains a challenge [19]. Several studies showed a high prevalence of cough (40%-45%) among patients with ZD regardless of the diverticulum size [6,10,15,20]. The presence of cough in patients with ZD can be explained by the response of cough receptors to the pressure stimuli caused by the diverticulum and the inhaled particles. Hence, surgical intervention of ZD can result in an effective reduction of cough and the burden of respiratory symptoms and complications [21]. Our data add to the literature not only because cough was present in almost two-thirds of our patients with ZD as a symptom but also because we noted that the presence of cough was associated with patients' decision to undergo surgical repair.

Previous studies by Resouly et al. and Hunt et al. described that dysphonia is usually more frequent among patients with a small-sized pharyngeal pouch [22,23]. Our results not only correlate with their findings but also suggest a negative impact of the presence of dysphonia when deciding whether or not to undergo ZD surgical repair. If dysphonia is the only symptom, a comprehensive voice evaluation should be completed to evaluate for other contributing factors. Other studies have also reported that patients with dysphagia present with significantly greater severity of breathing dysfunction and higher scores in voice-related questionnaires, including the Voice Handicap Index (VHI)-10, Glottal Function Index (GFI), and Reflux Symptom Index (RSI) [24-26]. We consider that our findings suggest underlying pathophysiology between dysphagia and dysphonia in the context of a ZD diagnosis, and that has yet to be elucidated and evaluated in further prospective studies. Although our patients with dysphonia were assessed by a laryngologist as part of the standard of care during ZD assessment, one of our study's limitations is the lack of voice-related questionnaire data, limiting an objective analysis regarding this phenomenon.

Our study's approach involves preoperative symptoms of ZD due to their impact on the quality of life. Several studies have correlated the surgical repair of the diverticula with the resolution of symptoms and a positive impact on patients' life [10,13,20,27]. Therefore, we consider that a clear explanation of symptoms' etiology by the clinician could improve the likelihood of the patient's decision to undergo surgical repair [28,29]. The patient and physician make an educated decision based on a clear understanding of the patient's symptoms. Prognosis with or without surgery should be discussed. The challenge lies in choosing the appropriate procedure in any given case. An early surgical repair of ZD even in patients with a small diverticulum or mild symptoms will prevent a nutritional compromise and potential escalating comorbidity over time [8]. It has been reported that an appropriate approach to patients' concerns contributes to higher satisfaction levels once the postoperative clinical improvement is evident. For example, in 2018, Tabola et al. evaluated a cohort of 44 patients with large ZD who underwent surgical repair. They found 100% postoperative satisfaction, even in those patients who developed surgical complications, answering that they would choose the operation again under the same postoperative results [13]. Bonavina et al. found a high satisfaction rate after ZD repair in a cohort of 100 patients with dysphagia and respiratory symptoms [27]. Palmer et al. reported resolving of postprandial cough as part of the achieved patient-referred goals after ZD repair [20]. Greene et al. confirmed that the resolution of swallowing dysfunction-related symptoms is excellent after the surgical approach, resulting in recovering quality of life [10]. We understand that dysphagia, cough, and dysphonia are complex and interrelated dysfunctions that can be caused by comorbidities [30]; however, our study highlighted their contribution to the surgical repair process. Although some patients are asymptomatic and diverticula are incidental findings, most patients are symptomatic. Dysphagia, regurgitation, and pain are common complaints; however, symptoms are often nonspecific and may be the result of another disorder. The possibility of ZD presence should be considered in clinical practice, not only due to the severe consequences but also due to the existence of effective therapeutic methods when diagnosed. A discussion regarding the risk/benefit ratio and postoperative results is imperative.

As a retrospective study, some intrinsic limitations have to be considered when analyzing our results. For example, co-interventions and confounders could not be controlled due to the nature of our study. Additionally, there was limited data in some medical records, such as clinical notes regarding the symptoms' improvement after the ZD surgical repair and voice-related questionnaires. To confirm our findings, studies involving the preoperative assessment of patient-reported outcome measures, including the VHI-10, GFI, RSI, and Voice-Related Quality of Life (VRQOL) score, are needed. Further studies that evaluate the decision-making of patients with ZD will contribute to more robust evidence to establish strategies to improve patients' quality of life and postoperative satisfaction.

## Conclusions

The patients' final decision to undergo surgery usually involves interrelated factors, including symptomatic burden, comorbidities, and recommendation of the surgeon. This study has identified that diverticula size and the presence of symptoms such as dysphagia, cough, and dysphonia are significant factors influencing decision-making for surgical repair in patients with ZD. The results can be used to improve clinical insight in treatment planning and pre- and postoperative counseling. The patient and physician make an educated decision based on a clear understanding of the patient's symptoms. Prognosis with or without surgery should be discussed. The challenge lies in choosing the appropriate procedure in any given case. The goal is to not cause harm to the patient.
